# The mediating effect of after-midnight use of digital media devices on the association of internet-related addictive behavior and insomnia in adolescents

**DOI:** 10.3389/fpubh.2024.1422157

**Published:** 2024-07-11

**Authors:** Thomas Lederer-Hutsteiner, Kai W. Müller, Matthias Penker, Erwin Stolz, Elfriede R. Greimel, Wolfgang Freidl

**Affiliations:** ^1^Institute of Social Medicine and Epidemiology, Medical University of Graz, Graz, Austria; ^2^Outpatient Clinic for Behavioral Addiction, Department of Psychosomatic Medicine and Psychotherapy, University Medical Center, Johannes Gutenberg University Mainz, Mainz, Germany; ^3^Center for Social Research, University of Graz, Graz, Austria; ^4^Department of Obstetrics and Gynaecology, Medical University of Graz, Graz, Austria

**Keywords:** insomnia, internet-related addictive behavior, late night use of digital media devices, adolescents, prevention

## Abstract

**Background:**

There is evidence that overexposure to digital media devices (DMD) can not only lead to addictive patterns of internet use, but also cause insomnia symptoms. The aim of this cross-sectional study among adolescents is to provide an estimate of the prevalence of sleep impairments and to explore the mediating role of after-midnight use of DMD between internet-related addictive behavior (IRAB) and insomnia.

**Methods:**

2,712 school students from Styrian schools participated in a population-representative online survey in a supervised school setting in spring 2022. School students were screened using established and validated scales. Data analysis was carried out using multiple imputation, linear multilevel regression and mediation analysis.

**Results:**

Prevalence estimation indicates high proportions of clinically relevant moderate [12.6% (11.3%; 14.1%)] and severe [3.6% (2.9%; 4.4%)] insomnia, with an additional 30.6% (29.0%; 32.2%) at subthreshold level. DMD are typically used after midnight an average of 1.66 (1.58; 1.75) evenings with subsequent school day per school week. Linear multilevel regression analysis shows significant associations for sleep disparities as outcome variable e.g., with generalized anxiety [*b* = 0.329 (0.287; 0.371)], after-midnight use of DMD [b = 0.470 (0.369; 0.572)] and IRAB [*b* = 0.131 (0.097; 0.165)]. Mediation analysis shows a mediated proportion of 18.2% (13.0%; 25.0%) of the association of IRAB and insomnia by after-midnight use of DMD [Indirect effect: *b* = 0.032 (0.023; 0.040), direct effect: *b* = 0.127 (0.083; 0.170)].

**Conclusions:**

Although the cross-sectional nature of this study limits causal inference, the results indicate a need for policies, which are already in preparation in Styria as part of a respective action plan.

## 1 Introduction

Since the introduction of smartphones, which provide almost unrestricted access to internet-based applications anytime and anywhere, there has been a continuous increase in the frequency and duration of using DMD and online applications. According to the Austrian Internet Monitor, in 1996 only 2% of Austrians aged 14 and above used the internet daily, whereas this percentage rose to 43% in 2007 with the launch of the iPhone, the precursor of today's mobile internet use enabling smartphones, and had reached 82% in 2022. Among young people aged 14–19 years, this percentage is shown to be even higher at 91% in 2022 (1). The JIM study, conducted in Germany since 1998 among 12–19 year-olds also shows a similar trend, indicating usage times of at least 4 h daily for leisure-related activities within this target group in 2022 (2). Without doubt, these devices offer countless advantages and help to facilitate daily concerns if used in a supportive manner.

Not only, but especially among adolescents, the supporting function of using DMD and internet applications they access, has increasingly been replaced by a day-dominating one, which in some cases even turn into addictive usage. Since its first appearance in scientific context in 1998 (3) the maladaptive and addictive use of DMD and the internet, commonly conceptualized and referred to as Internet Addiction or Internet Use Disorder, has been increasingly issued in the last two decades. The American Psychiatric Association and the World Health Organization have included this behavioral phenomenon in their classification manuals Diagnostic and Statistical Manual of Mental Disorders (DSM-5; since 2013 as a condition for further study) and forthcoming International Classification of Diseases (ICD-11; in the chapter “Disorders due to behavioral addictions”), respectively. Criticism for this decision has been raised by several authors due to lacking conceptual and empirical foundations (4–6). However, addictive use of DMD and the internet has been the subject of numerous epidemiological and interventional studies worldwide. A recent meta-analysis reported a pooled prevalence of this phenomenon of 7%, based on 113 epidemiological studies involving almost 700,000 individuals from 31 countries, with prevalences from single studies ranging between 10 and 20% (7). Adolescents and young adults are consistently identified as the target group with the highest prevalences in almost all studies. It should be noted that this meta-analysis' single studies did not reflect the restrictions due to the COVID-19 pandemic in many countries and the associated intensified usage. It is therefore likely that there has been a further increase in prevalence in certain target groups over the course of the pandemic as shown for example in Germany (8, 9).

Regardless of whether adolescents' DMD usage is addictive or not, it is discussed that it has developed into a widespread pre-sleep routine (10). Pre-sleep and late night use of DMD account for delayed bedtime and hence also shorter sleep duration (11), at least during weekday evenings due to the fixed school schedule. Austrian schools, for example, start at 8 a.m. at the latest. Screen-based pre-sleep activities and their effects on sleep outcomes have been investigated in many studies. Systematic literature reviews show adverse associations of screen-based pre-sleep activities and sleep outcomes, reducing both the quantity as well as the quality of sleep (11–14). Since the majority of the included observational studies in theses reviews were cross-sectional, it is difficult to infer the causal relationship between digital media use and sleep outcomes. A recent meta-analysis focusing exclusively on longitudinal studies (15) showed a significant negative effect of utilization time of digital media at one time point on sleep health on a later time point but not vice versa. Conversely, sleep health at one time point was significantly negatively associated with dysfunctional use of digital media on a later time point. Another meta-analysis, focusing on studies that examined the relationship between Internet Addiction and sleep problems, shows that people with Internet Addiction are 2.4 more likely to have sleep problems than people who are not internet addicted (based on studies using standardized measures of sleep problems). Besides, this meta-analysis also showed shorter sleep duration (pooled standardized mean difference = −0.24 h) (16). Other meta-analyses also showed a significant positive correlation between mobile phone addiction and poor sleep quality (17) and an increased risk of adolescents with mobile phone addiction having sleep disorders (18). Additionally, Internet Addiction was found to be positively related to late-night internet use (19). In summary, the mentioned studies provided evidence that IRAB as well as late-night internet use are linked to impaired sleep and that also IRAB is linked to late-night internet use. However, to the best of the authors' knowledge no study has been published, that has examined the combined relationship between these three variables, so it remains unclear whether the relationship between addictive usage and adverse sleep outcomes is mediated by late night usage.

Parental rules could provide a framework for adolescents' use of the internet and DMD, limiting its overall extent generally, but also its occurrence at bedtime. Available studies show inconsistent findings. Reduced time spent online was significantly associated with restrictive parental mediation (parental regulative rules regarding their children's internet use) in a Korean study among children from fourth to ninth grade and their parents (20). The effect was more pronounced in children with low self-control. However, restrictive mediation was not associated with children's addictive use. A weak negative relationship between excessive internet use and general parenting styles such as warmth, control and authoritative parenting has been carried out in a recent meta-analysis (21), suggesting that parental behavior generally matters regarding the issue of internet use. On the other hand the meta-analysis also yielded, that there was no overall association between excessive internet use and respective restrictive parental mediation. For older adolescents this association was even statistically significantly positive. In contrast, a large scale study in a sample of about 19,000 adolescents aged 11–16 years in 25 European countries, yielded a significant association between excessive internet use and restrictive parental mediation (22). Regarding the effects of parental mediation on DMD usage before bedtime and sleep outcomes, Buxton et al. (23) conclude their findings in a study in households with a child aged 6–17 years as: “*Children generally have better age-appropriate sleep in the presence of household rules and regular sleep-wake routines*.” It therefore seems plausible to assume, that parental rules affect the association between IRAB and late night use of DMD.

According to the expert panel of the American Academy of Sleep Medicine adolescents should on average have 8–10 h of sleep per night to maintain health (24, 25). A recent large scale study among 252,195 Australian pre- (8–11 years), early (12–14 years) and late adolescents (15–18 years) shows for all cohorts that receiving < 8 h of sleep on most nights was significantly predicted by mobile phone use at night. The chance of sleeping more than 8 h on most nights when using a mobile phone at night at least once a week was about 50% less (aOR_Early adolescent_ = 0.46. aOR_Late adolescent_ = 0.54. aOR_Pre − adolescent_ = 0.56) compared to no night-time use. In all cohorts females reported more often not to receive 8 h of sleep than males (26). The chance of receiving the recommended 8 h of sleep is very low, if DMD are used after midnight due to the aforementioned school start in Austria. Midnight can therefore be considered an interesting reference point for operationalising late-night use. (In)Sufficient sleep is closely related to mental health. One of the few longitudinal studies on this issue was conducted among a sample of 4,494 US-adolescence with 6- to 7-year follow-up. On a cross-sectional level insomnia symptoms were significantly associated with use of alcohol and drugs, depression, suicide ideation and suicide attempts. Moreover, on a prospective level adolescents' insomnia symptoms were found to be a significant risk factor (OR = 2.3) for being diagnosed with depression in young adulthood (27). In line, a recent meta-analysis concludes, that sleep disturbances such as insufficient sleep, circadian rhythm disturbances or insomnia significantly foster the development of mood or psychotic disorders (OR = 1.9) (28). Of course, sleep disturbances are not only the cause of, but might also result from mental health problems. For example anxiety problems may both prolong but also result from sleep problems and therefore act in a bidirectional relationship as a cause, but also as a consequence of insufficient sleep (29, 30). Insomnia symptoms tend to persist during adolescence with low odds of a full remission (31). A recent publication of a cohort study with 2-year follow-up showed that insufficient sleep acts as a serious long-term risk factor for neurocognitive development in early adolescence. The authors “*… highlight the value of early sleep intervention to improve early adolescents' long-term developmental outcomes”* (32). Additional long-term consequences include, for example, cardiovascular disease, metabolic syndrome, type 2 diabetes mellitus (33). To prevent chronification and detrimental health outcomes early identification of insomnia symptoms is important (34). Target groups with higher vulnerability for insomnia symptoms are for example girls, children of low socioeconomic status, adolescents with existing psychological, neurological or metabolic problems and those with an evening chronotype (31, 35).

## 2 Research questions

**Figure 1:**
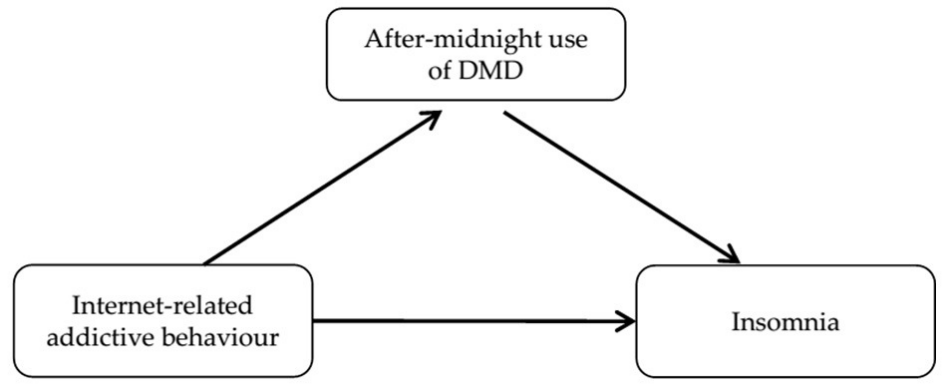
Conceptual model of research question 3. Simple mediation model adjusted by school classes, anxiety symptoms, overall psychosocial constitution, age, gender, and socioeconomic status.

Population-representative data on the prevalence of insomnia symptoms and the extent of overexposed as well as late-night use of DMD among school students is scarce, particularly in Austria. Closing this gap will support evidence-based decision-making in the context of target group-specific policy planning. Furthermore, by examining the relationship between insomnia, IRAB and after-midnight use of DMD as a potential mediator of the association between IRAB and insomnia, we address an unexplored aspect of research on insomnia in the context of IRAB. Hence, the aim of this article is to provide answers to the following questions:

1. How many school students show sleep problems and how are they associated with sociodemographic and mental health characteristics, as well as usage behavior of DMD?
2. What is the extent of DMD use at bedtime (as a pre-sleep activity and after-midnight, respectively) and is after-midnight use of DMD associated with sociodemographic and mental health characteristics, as well as usage behavior of DMD?
3. If IRAB is associated with sleep problems, how is this effect mediated by after-midnight use of DMD (see Figure 1)?

## 3 Materials and methods

The data used in this publication originate from a commissioned work assigned by Gesundheitsfonds Steiermark as the client and the social research company x-sample as the contractor to estimate the prevalence of IRAB among school students in Styria, Austria. Usage of the data for this publication has been approved by Gesundheitsfonds Steiermark.

### 3.1 Study design and addressed population

Data was collected with a population-representative, cross-sectional survey. The addressed population were school students in Styrian schools of all school types (extra occupationally forms were excluded) from grade 7 (with 12 years as the minimum age) up to grade 13 (with 19 years as the regular maximum age; in case of repeaters or school students in vocational schools age can be even higher, so a cut-off of 25 years was set). Included school types were pre-vocational year, vocational school, new secondary school, academic secondary school, higher technical and vocational colleges and intermediate technical and vocational schools. Given this definition, population data was obtained from Statistics Austria for all 3,537 classes, which met these criteria. Class-based information contained school name, class name, school type, region, grade and totals of female and male school students in the respective class.

### 3.2 Sampling

Based on the described population data a stratified one stage clustered random sampling procedure with classes as selection units was applied. The clustered sample was stratified to control for region, school type and grade and also balanced for gender. Based on an intended sample size of 2,000 school students and under the assumptions that 50% of the drawn school classes would also participate and that the classes consist of an average of 20 school students, 201 classes from 116 schools were randomly sampled.

### 3.3 Data collection process

The Styrian directorate of education provided a letter of approval and also sent an announcement letter to all sampled schools to explicitly support this survey. Based on this framework all school principals were contacted by phone to provide detailed information about the background and conduction of the survey. 103 schools with a total of 179 sampled classes agreed to participate in the survey. Only 13 schools did not participate, mostly because of limited resources due to the COVID-19 pandemic or because of too many survey requests. However, four schools have offered nine additional participating classes for the survey study, finally resulting in a total of 188 classes from 103 schools. On March, 22nd 2022 the school principals received documents with all the information (e.g., login data, parents' information letter, study outline) needed to conduct the survey independently. One week after sending the envelopes all principals were asked to confirm the receipt. Only a few did not receive the envelopes. In these cases the documents were re-sent via e-mail. The survey was conducted online using the platform www.onlineumfragen.com in schools during a school hour. The implementation deadline was set at May, 27th 2022. Teachers were able to choose a time point between March, 22nd and May, 27th 2022 that was convenient for them and the school students to complete the survey. Teachers have been instructed to survey all school students of the sampled classes and to exclude all school students who were at home due to illness or other reasons. Within the survey period all classes have been taught in presence modus, no class has been in distance learning. At the beginning of the questionnaire each school student had to provide informed consent, in order to proceed with the survey, i.e., participation was voluntary. If no consent has been given, the survey was closed.

### 3.4 Response rate

Out of the 201 randomly sampled school classes, 175 completed the survey and provided data. The class-based response rate was 87.1%, which can be considered to be very good.

### 3.5 Data cleaning procedures

**Figure 2:**
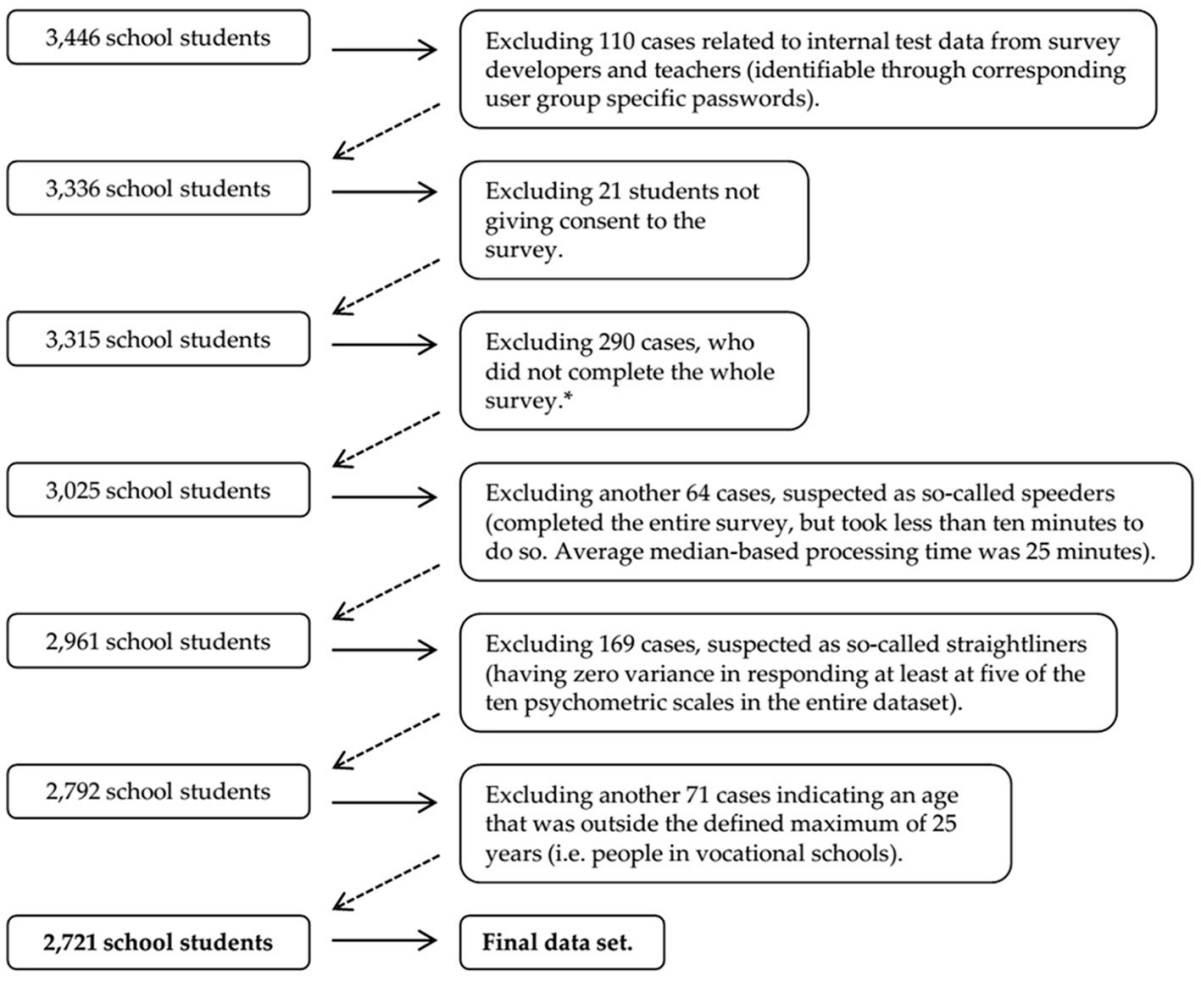
Flow chart of the data cleaning process. *School students who did not complete the whole survey do not differ from those who did in terms of insomnia, IRAB and after-midnight use of DMD as the major variables of the mediation model [Insomnia: T(2,807) = 0,570; *p* = 0.569. IRAB: T(3,131) = 0.213; *p* = 0.831]. After-midnight use of DMD: T(2,992) = −0.177; *p* = 0.859.

The original data set contained a total of 3,446 school students. The following data cleaning procedures have been applied in order to ensure the best possible data quality, resulting in a final analytical total sample size of 2,721 school students (see Figure 2).

### 3.6 Measures and classification

The main dependent variable in this study is insomnia. Further variables involved either as predictor, mediator or covariate are IRAB, DMD use as a pre-sleep activity, after-midnight use of DMD, age, gender, socioeconomic status, parental rules, generalized anxiety and overall psychosocial constitution. All measures were integrated in the online-questionnaire submitted. In advance a draft of the questionnaire was subjected to a pretest with seven school students aged 13–17 years in individual setting, another 23 school students in group setting (an entire class of grade 7, aged 13 years) and a teacher of the German language. Pretests aimed to ensure comprehensibility and to assess processing time. Based on minimal adjustments to the wording of individual items the questionnaire was approved for data collection with a final set of 141 individual items. The median-based processing time was 25 min.

#### 3.6.1 Insomnia

Insomnia was measured using the self-reporting format of the German translation of the Insomnia Severity Index (ISI) (36), designed for assessing sleep problems. On 5-point Likert-scales (scoring from 0 to 4) school students rated the extent of impairments of falling asleep, staying asleep and waking up too early, their satisfaction with their sleep, levels of distress and concern as well as interference with daily living (performance and wellbeing) caused by their sleep in the last 2 weeks. Originally ISI consists of seven items. The item (“How noticeable to others do you think is your sleep problem in terms of impairing the quality of your life?”) has been skipped as pretests has shown that school students had difficulties in understanding this question yielding multiple interpretations. The scores of the remaining six items were summed with higher scores indicating a higher severity of insomnia (scores ranging from 0 to 24). Sum scores were only calculated for participants with valid responses to all six items. The internal consistency in the present study is acceptable (α = 0.79, ω = 0.79). Classification is based on cut-off scores that have been validated by Gerber et al. (36), taking into account the reduced maximum sum score due to skipping one item. Cut-off scores of insomnia grades were adjusted proportionally to the reduced maximum score. Classification was carried out as “no clinically significant insomnia” (score from 0 to 6), “subthreshold insomnia” (score from 7 to 12), “clinically significant moderate insomnia” (score from 13 to 18) and “clinically significant severe insomnia” (score from 19 to 24).

#### 3.6.2 Internet-related addictive behavior

IRAB was measured using the German self-reporting short form of the Compulsive Internet Use Scale (CIUS) (37) and its validation (38). CIUS is based on diagnostic criteria for dependence and behavioral addictions such as Pathological Gambling embedded in DSM-IV. The original form of CIUS (39) consists of 14 items; the short version used here consists of 7 items including items 1, 3, 5, 7, 9, 11, and 12 of the original 14-items scale. On 5-point Likert-scales (scoring from 0 = “never” to 4 = “very often”) school students rated loss of control, inter- and intrapersonal conflict, mental preoccupation and internet-related coping with unpleasant mood. The scores of all seven items were summed with higher scores indicating a higher severity of IRAB (scores ranging from 0 to 28). Sum scores were only calculated for participants with valid responses to all seven items. The internal consistency in the present study is good (α = 0.82, ω = 0.82). Classification is based on a validated cut-off score (38). For the purpose of reducing false positive assignments, the value 13 was chosen as cut-off, showing high specificity of 0.97 in the validation study by Besser et al. Classification was carried out as “No noticeable IRAB” (score from 0 to 12) and “IRAB” (score from 13 to 28).

#### 3.6.3 After-midnight use of DMD on evenings with subsequent school days

School students were asked “If you have school the next day, how often do you usually use one of these devices after midnight?” The term “devices” was specified as “internet-enabling smartphones, tablets, gaming consoles, computers, or other digital devices”. Participants could indicate, if usage after midnight occurs on 0, 1, 2, 3, 4, or 5 of 5 such days. Thus, scores range from 0 to 5, with higher scores indicating a higher extent of after-midnight use on subsequent school days. Midnight as reference point was chosen to infer, how many pupils were clearly unable to get the recommended 8 h of sleep (25), as schools in Austria start at 8 a.m. at the latest.

#### 3.6.4 Use of DMD as a pre-sleep activity on evenings with subsequent school days

The format and assignment of values was the same as in the inquiry of after-midnight use (see above), but with different question: “If you have school the next day, how often do you usually use one of these devices directly before falling asleep?”

#### 3.6.5 Age

Age was assessed by asking “Your age?” and school students could enter their age in an open format.

#### 3.6.6 Gender

Gender was assessed by asking “Your gender?” and providing “Male”, “Female” and “Different gender” as response options. All those who selected “Different gender” could specify the respective gender definition in their own words.

#### 3.6.7 Socioeconomic status

Classification of socioeconomic status (SES) is based on educational background of school students' mother and father, respectively (5-point scale with higher values indicating higher formal education) and school students' assessment of parents' available financial resources (5-point scale with higher values indicating higher financial resources). The index was calculated as a sum score of all indicators using just the highest value of parents' educational background (scores ranging from 0 to 8). Sum scores were only calculated if valid information on all indicators was available.

#### 3.6.8 Parental rules

Parental rules were assessed in a unidimensional way by simply asking “Did you parents establish rules about how much time you are allowed to use DMD in your leisure time?” and school students could choose between “Yes” (coded with 1) or “No” (coded with 0).

#### 3.6.9 Generalized anxiety

Generalized anxiety was measured using the German self-reporting form of the Generalized Anxiety Disorder Screener (GAD-7) (40) and its validation in a sample of pediatric patients with anxiety disorder (confirmed by structured interviews) (41). GAD-7 is based on diagnostic criteria for Generalized Anxiety Disorder embedded in DSM-IV and assesses the severity of anxiety symptoms. GAD-7 consists of 7 items, which are self-rated on 4-point Likert-scales (0 = “Not at all,” 1 = “Several days,” 2 = “More than half the days,” and 3 = “Nearly every day”). School students rated (1) “Feeling nervous, anxious or on edge,” (2) “Not being able to stop or control worrying,” (3) “Worrying too much about different things,” (4) “Trouble relaxing,” (5) “Being so restless that it is hard to sit still,” (6) “Becoming easily annoyed or irritable,” and (7) “Feeling afraid as if something awful might happen”. The scores of all seven items were summed with higher scores indicating a higher severity of anxiety disorder (scores ranging from 0 to 21). Sum scores were only calculated for participants with valid responses to all 7 items. The internal consistency in the present study is excellent (α = 0.90, ω = 0.90). Classification is based on a cut-off score of 11, which showed the best compromise between sensitivity (0.97) and specificity (1.00) as suggested by Mossman et al. (41). Two groups were differentiated: “no noticeable anxiety disorder” (score from 0 to 10) and “at least moderate anxiety disorder” (score from 11 to 21).

#### 3.6.10 Overall psychosocial constitution

Overall psychosocial constitution was measured using the validated German self-reporting form of the Strength and Difficulties Questionnaire (SDQ) (42). SDQ measures young people's internalizing and behavioral problems and is valid and widely used to screen for mental health problems (42). SDQ consists of 25 items, which are to be self-rated on 3-point -scales (0 = “Not true,” 1 = “Somewhat true,” and 2 = “Certainly true”). School students rated items along the five subscales emotional symptoms, conduct problems, hyperactivity/inattention, peer relationship problems and prosocial behavior, each consisting of five items. Subscale scores were calculated by summing the respective item scores (some of them have been inverted beforehand). To calculate the total difficulties index, the scores of all subscales except prosocial behavior were summed with higher scores indicating a higher severity of mental health problems (scores ranging from 0 to 40). Sum scores were only calculated for participants with valid responses to all 20 items. The internal consistency in the present study is acceptable (α = 0.78, ω = 0.77). Classification was carried out as “Normal” (scores from 0 to 15), “Borderline” (scores from 16 to 19), and “Abnormal” (scores from 20 to 40).

### 3.7 Statistical analysis

Prevalence estimates in research questions 1 and 2 have been carried out by applying calibrating weights to account for sample loss and the associated mismatch between population and sample structure. Calibration weights were calculated by incorporating combinations of region, school type, grade and gender as stratas (minimal weight 0.60, maximum weight 3.50). Due to a high cumulative rate of 36.35% of cases with at least one missing value, multiple imputation (*m* = 40) using fully conditional specification (FCS) and predictive mean matching (Multiple Imputation Module of IBM SPSS Version 29) was conducted based on all variables (on item-level in case of scales) included in the regression models to maintain statistical power and minimize potential selection bias. To account for the nesting of school students in classes, resulting in potential dependencies of observations of students within the same class, a multilevel approach with random intercepts (Linear Mixed Models Module of IBM SPSS Version 29) has been used. The assumptions of regression analysis were tested and found to be met. Research question 3 was addressed with a mediation analysis with IRAB as predictor, extent of after-midnight use of DMD as mediator and insomnia as outcome variable. Mediation analysis was adjusted by school classes, generalized anxiety, overall psychosocial constitution, age, gender and socioeconomic status (see Figure 1) and performed using the mediation R-package (43).

## 4 Results

### 4.1 Sample's sociodemographic characteristics

The population-adjusted weighted sample consists of 2,712 participants, 48.4% are males, 44.4% females and 4.4% school students, who assigned themselves a different gender (see Table 1). 2.8% of the responses on gender were undisclosed. School students are on average 15.77 years of age (SD = 2.20; MIN = 12; MAX = 25). Most of the school students attend academic secondary school (27.3%), followed by higher technical and vocational colleges or intermediate technical and vocational schools (26.4%), vocational schools (23.6%), new secondary schools (20.0%) and prevocational year (2.7%). Most of the school students attend schools in the central region of Styria, the province's capital Graz and suburbs (39.0%), followed by East-Styria (17.1%), Southwest-Styria (16.5%), Southeast-Styria (11.8%), Upper-Styria West (6.5%), Upper-Styria East (5.4%), and Liezen (3.6%).

**Table 1:** Weighted sample's sociodemographic characteristics.

	N^*^(%)	**Mean (SD)**
**Gender**		
Male	1,318 (48.4)	
Female	1,207 (44.4)	
Different gender	121 (4.4)	
*Undisclosed*	75 (2.8)	
**Age**		15.77 (2.20)
*Undisclosed*	98 (3.6)	
**School types**		
New secondary school	544 (20.0)	
Academic secondary school	744 (27.3)	
Prevocational year	73 (2.7)	
Vocational school	642 (23.6)	
HTVC or ITVC^**^	719 (26.4)	
*Undisclosed*	0 (0.0)	
**Educational region of attended school**		
Liezen	98 (3.6)	
Upper-Styria East	148 (5.4)	
Upper-Styria West	177 (6.5)	
East-Styria	466 (17.1)	
Central region of Styria	1,060 (39.0)	
Southeast-Styria	321 (11.8)	
Southwest-Styria	450 (16.5)	
*Undisclosed*	0 (0.0)	

^*^Total sample size is 2,712 cases (unimputed data). Column sums of some variables differ slightly due to roundoff issues associated with weighting.

^**^Higher technical and vocational colleges or intermediate technical and vocational schools.

### 4.2 Research question 1: prevalence of insomnia symptoms and correlates

According to Insomnia Severity Index-screening 46.8% of the entire sample show sleep problems, 30.6% (95%-CI: 29.0–32.2) on a subthreshold, 12.6% (95%-CI: 11.3–14.1) on a clinically relevant moderate and 3.6% (95%-CI: 2.9–4.4) on a severe level (see Table 2).

**Table 2:** Weighted sample's descriptive statistics of outcomes, predictors and covariates.

	*N* (%)^*^	**Mean (SD); median (IQR)**	**95%-CI**
**Insomnia**		7.51 (5.22); 7.00 (8.00)	7.27–7.75%
No	1,232 (45.3)		43.2–47.3%
Subthreshold	832 (30.6)		29.0–32.2%
Moderate	343 (12.6)		11.3–14.1%
Severe	97 (3.6)		2.9–4.4%
*Undisclosed*	216 (7.9)		6.6–9.5%
**Using DMD as a pre-sleep activity …** ^**^		4.22 (1.42); 5.00 (1.00)	4.16–4.29
Usually 0 out of 5 days	126 (4.6)		3.9–5.6%
Usually 1 out of 5 days	91 (3.4)		2.7–4.1%
Usually 2 out of 5 days	130 (4.8)		4.0–5.7%
Usually 3 out of 5 days	214 (7.9)		6.9–8.9%
Usually 4 out of 5 days	231 (8.5)		7.4–9.8%
Usually 5 out of 5 days	1.848 (67.9)		65.8–69.9%
*Undisclosed*	81 (3.0)		2.4–3.7%
**Using DMD after midnight …** ^**^		1.66 (1.82); 1.00 (3.00)	1.58–1.75
Usually 0 out of 5 days	1.044 (38.4)		36.1–40.7%
Usually 1 out of 5 days	390 (14.4)		13.0–15.8%
Usually 2 out of 5 days	333 (12.2)		11.1–13.4%
Usually 3 out of 5 days	273 (10.0)		9.0–11.2%
Usually 4 out of 5 days	119 (4.4)		3.7–5.1%
Usually 5 out of 5 days	372 (13.7)		12.3–15.2%
Undisclosed	190 (7.0)		6.0–8.1%
**Socioeconomic status**		6.11 (1.50); 6.00 (2.00)	6.03–6.18
*Undisclosed*	574 (21.1)		19.2–23.1%
**Parental rules**			
No	2,164 (79.5)		77.4–81.5%
Yes	476 (17.5)		15.6–19.6%
*Undisclosed*	81 (3.0)		2.4–3.7%
**IRAB**		10.02 (5.71); 10.00 (8.00)	9.75–10.29
No	1,841 (67.7)		65.7–69.6%
Yes	879 (32.3)		30.4–34.3%
*Undisclosed*	1 (0.0)		0.0–0.2%
**Generalized anxiety**		6.87 (5.52); 6.00 (8.00)	6.57–7.17
No	1,790 (65.8)		63.3–68.1%
Yes	647 (23.8)		21.8–25.8%
*Undisclosed*	285 (10.5)		8.9–12.3%
**Overall Psychosocial constitution**		13.95 (6.07); 13.00 (9.00)	13.61–14.30
Normal	1,413 (51.9)		49.5–54.4%
Borderline	433 (15.9)		14.6–17.3%
Abnormal	450 (16.5)		15.0–18.1%
*Undisclosed*	426 (15.7)		13.7–17.8%

^*^Total sample size is 2,712 cases (unimputed data). Column sums of some variables differ slightly due to roundoff issues associated with weighting.

^**^… on evenings with subsequent school day (per week).

Table 3 shows the results of the multilevel regression model of insomnia. 36.4% of the total variance can be explained by fixed predictors (*R*^2^ marginal) with very little additional contribution in variance explanation due to school classes as level 2-random effect variable (*R*^2^ conditional). Thus, the extent of insomnia was homogeneous across school classes.

**Table 3:** Multilevel regression analysis with insomnia symptoms as dependent variable (Level 1: school students. Level 2: school classes).

	***b* (SE); (95%-CI)**	**β**
**Fixed effects**		
Intercept	1.141(0.905); (−0.633; 2.915)	−0.032
IRAB	0.131 (0.017)^***^ (0.097; 0.165)	0.142
PRESLP	0.089 (0.066) (−0.040; 0.219)	0.023
AFTMIDNGT	0.470 (0.052)^***^ (0.369; 0.572)	0.163
RULES	0.335 (0.245) (−0.145; 0.815)	0.032
GAD	0.329 (0.021)^***^ (0.287; 0.371)	0.346
PSYCONST	0.089 (0.020)^***^ (0.051; 0.127)	0.103
AGE	0.097 (0.042)^*^ (0.014; 0.181)	0.041
SES	−0.242 (0.061)^***^ (−0.361; −0.123)	−0.070
GENDER female	0.561 (0.182)^**^ (0.204; 0.918)	0.054
GENDER different	0.116 (0.415) (−0.698; 0.930)	0.011
**Random effects**	Pooled variance estimate	
Variance of class-intercept	0.236 (0.150); (−0.059; 0.531)	

IRAB, Internet-related addictive behavior symptoms (CIUS-score); PRESLP, Count of days using DMD as a pre-sleep activity on days with subsequent school day (per school week); AFTMIDNGT, Count of days on using DMD after midnight on evenings with subsequent school day (per school week); GAD, Generalized anxiety disorder symptoms (GAD-score); PSYCONST, Overall psychosocial constitution (SDQ-score); RULES, Parental regulative rules on the amount of using DMD (coded 0 = no and 1 = yes); AGE, School students' age; SES, Socioeconomic status score; GENDER: Dummy-coded gender with male as reference.

*b*, Pooled unstandardized coefficient; SE, Pooled standard error; 95%-CI, Pooled 95%-confidence interval of *b*; β, Pooled standardized coefficient [standardization was carried out by dividing numeric variables by two standard deviations for a better comparison with binary variables (44)]. *N* = 2.721 (imputed data; *m* = 40); pooled variance estimate of Level 1-residuals = 17.246; pooled ICC adjusted/conditional = 0.013/0.009; pooled *R*^2^ marginal/conditional = 0.364/0.372; *R*^2^-values based on (45); ^*^p < 0.05. ^**^p < 0.01. ^***^p < 0.001.

Significant positive associations with sleep problems are indicated for anxiety problems showing the strongest relation, after midnight-use of DMD, IRAB, overall psychosocial constitution, being female (compared to male) and age. A significant negative association was found for socioeconomic status (the less, the more sleep problems). Using DMD as a pre-sleep activity, parental rules and assigning themselves a different gender are not associated with sleep problems.

**Figure 3:**
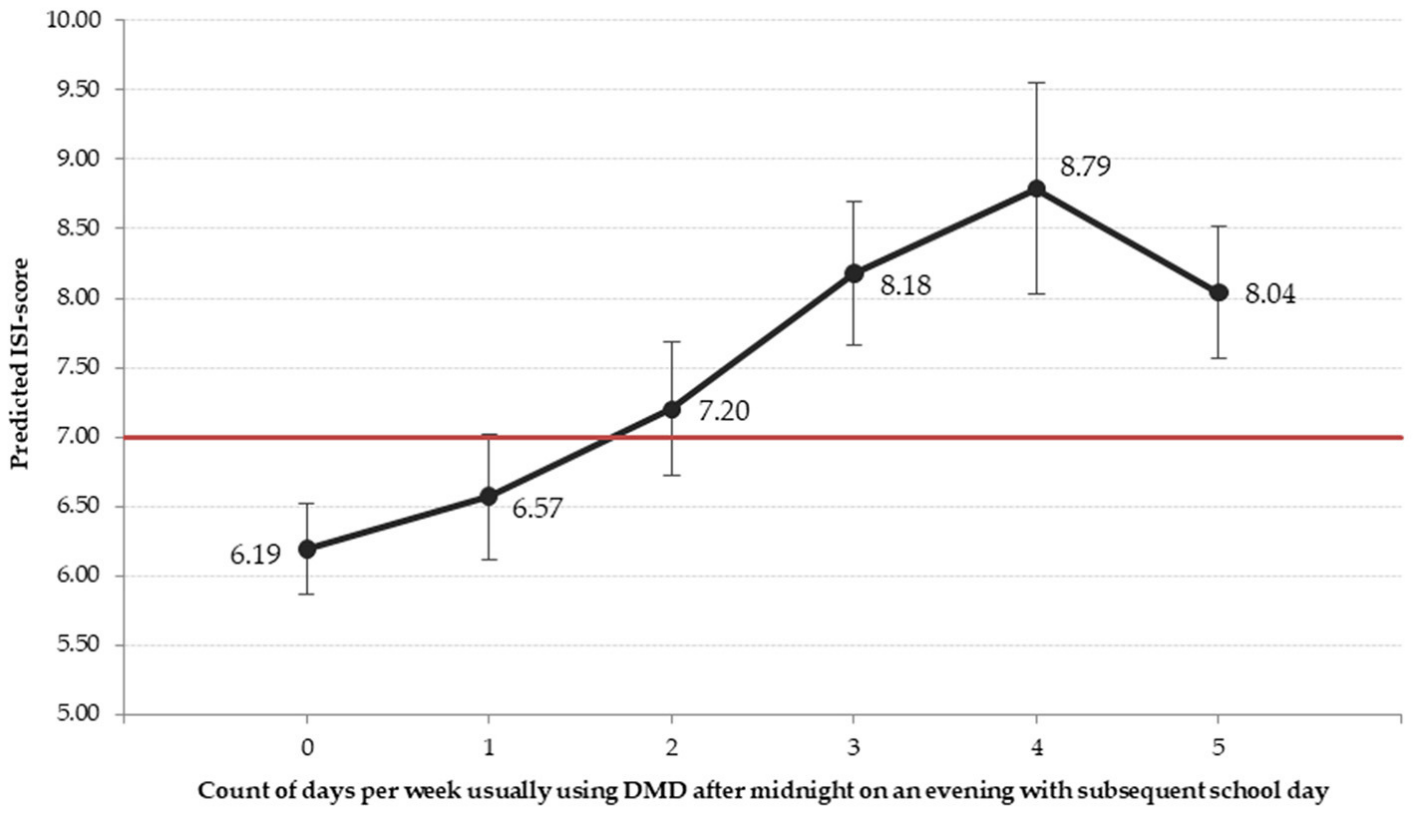
Predicted values of Insomnia Severity Index scale for count of days using DMD after midnight. ISI-score: Insomnia Severity Index score. The red line illustrates the cut-off for subthreshold insomnia (36). Prediction adjusted by all variables of the regression model in Table 3. The estimates are based on one of the 40 imputed data files. The results of the other files do not differ substantially and conclusions drawn are robust.

Figure 3 shows predicted values of Insomnia Severity Index based on the extent of using DMD after midnight and indicates a constant increase of insomnia as after-midnight use of DMD increases. Based on the point estimate, the average predicted value of 7, as an indication of switching into impaired sleep is exceeded by using DMD after midnight usually on more than one evening a week with subsequent school day. Taking the confidence interval into account this threshold is exceeded by respective usage on more than two evenings a week.

### 4.3 Research question 2: extent of after-midnight use of DMD and correlates

Table 2 shows, that 54.7% of the entire sample usually uses DMD after midnight on at least one of five evenings with subsequent school day per week. 13.7% (95%-CI: 12.3–15.2) usually use them on every single of such evenings. On average the examined school students usually use them on 1.66 of such evenings (SD: 1.82; 95%-CI: 1.58–1.75). 67.9% (95%-CI: 65.8–69.9) usually use DMD as a presleep activity on every single evening with subsequent school day. On average they usually use them on 4.22 of such evenings (SD: 1.42; 95%-CI: 4.16–4.29).

16.7% of the total variance of after-midnight use of DMD can be explained by the fixed predictor variables (*R*^2^ marginal) with little additional contribution in variance explanation due to school classes as level 2-random effect variable (*R*^2^ conditional; see Table 4). The extent of after-midnight use of DMD was therefore also homogeneous across school classes, although slightly more varied compared to sleep problems (see Table 3).

**Table 4:** Multilevel regression analysis with after-midnight use of DMD (on evenings with subsequent school day) as dependent variable (Level 1: school students. Level 2: school classes).

	**Model without interaction**	Model with interaction IRAB^*^RULES
	***b* (SE); (95%-CI); β**	***b* (SE); (95%-CI); β**
**Fixed effects**		
Intercept	−1.687(0.385)^***^ (−2.441; −0.932); 0.035	−1.758(0.388)^***^ (−2.519; −0.997); 0.035
IRAB	0.062(0.007)^***^ (0.049; 0.075); 0.195	0.066(0.007)^***^ (0.052; 0.079); 0.207
PRESLP	0.257(0.025)^***^ (0.208; 0.307); 0.196	0.260(0.025)^***^ (0.210; 0.309); 0.198
RULES	−0.172(0.094) (−0.357; 0.012); −0.048	0.085(0.190) (−0.287; 0.457); −0.044
IRAB^*^RULES		−0.025(0.016) (−0.055; 0.006); −0.077
GAD	0.014(0.008) (−0.002; 0.030); 0.043	0.014(0.008) (−0.002; 0.030); 0.043
PSYCONST	0.030(0.008)^***^ (0.015; 0.045); 0.100	0.030(0.008)^***^ (0.015; 0.045); 0.101
AGE	0.090(0.018)^***^ (0.054; 0.126); 0.111	0.091(0.018)^***^ (0.055; 0.127); 0.112
SES	−0.033(0.025) (−0.082; 0.016); −0.027	−0.033(0.023) (−0.083; 0.016); −0.028
GENDER female	−0.194(0.074)^**^ (−0.340; −0.048); −0.054	−0.195(0.074)^**^ (−0.341; −0.049); −0.054
GENDER different	0.336(0.166)^*^ (0.009; 0.662); 0.093	0.339(0.166)^*^ (0.012; 0.665); 0.093
**Random effects**	**Pooled variance estimate**	**Pooled variance estimate**
Variance of class-intercept	0.137(0.037) (0.065; 0.209)	0.137(0.037) (0.065; 0.209)

IRAB, Internet-related addictive behavior symptoms (CIUS-score); PRESLP, Count of days using DMD as a pre-sleep activity on days with subsequent school day (per school week); AFTMIDNGT, Count of days on using DMD after midnight on evenings with subsequent school day (per school week); GAD, Generalized anxiety disorder symptoms (GAD-score); PSYCONST, Overall psychosocial constitution (SDQ-score); RULES, Parental regulative rules on the amount of using DMD (coded 0 = no and 1 = yes); AGE, School students' age; SES, Socioeconomic status score; GENDER, Dummy-coded gender with male as reference.

*b*, Pooled unstandardized coefficient; SE, Pooled standard error; 95%-CI, Pooled 95%-confidence interval of *b*; β, Pooled standardized coefficient [standardization was carried out by dividing numeric variables by two standard deviations for a better comparison with binary variables (44)].

Model without interaction: *N* = 2.721 (imputed data; *m* = 40); pooled variance of Level 1-residuals = 2.581; pooled ICC adjusted/conditional = 0.050/0.042; pooled *R*^2^ marginal/conditional = 0.166/0.208; *R*^2^-values based on (45); pooled AIC/BIC = 10,454.440/10,466.250. ^*^p < 0.05. ^**^p < 0.01. ^***^p < 0.001.

Model with interaction: *N* = 2.721 (imputed data; *m* = 40); pooled variance of Level 1-residuals = 2.579; pooled ICC adjusted/conditional = 0.051/0.042; pooled *R*^2^ marginal/conditional = 0.167/0.209; *R*^2^-values based on (45); pooled AIC/BIC = 10,458.224/10,470.033. ^*^p < 0.05. ^**^p < 0.01. ^***^p < 0.001.

Likelihood Ratio Test: −2LL_Model without interaction_ = 10,450.440; −2LL_Model with interaction_ = 10,454.224; Diff_−2*LL*_ = 3.784; df = 1; *p* = 0.052.

Significant positive associations with after-midnight use of DMD are shown with IRAB, pre-sleep use of DMD, age, overall psychosocial constitution and gender (for those with different gender compared to male). Being female is negatively associated with after-midnight use of DMD (compared to male). Anxiety problems, parental rules and socioeconomic status are not associated with after-midnight use of DMD. Besides, IRAB and parental rules do not interact in their effect on after-midnight use of DMD.

### 4.4 Research question 3: mediation of the association between IRAB and insomnia symptoms

As IRAB was found associated with both after-midnight use of DMD and sleep problems and after-midnight use of DMD is associated with sleep problems, we calculated to what extent IRAB transmits its effect to sleep problems through the mediator after-midnight use of DMD. According to the non-significant interaction of IRAB and parental rules on their effect on after-midnight use of DMD (see Table 4), we excluded a respective potential moderation.

**Figure 4:**
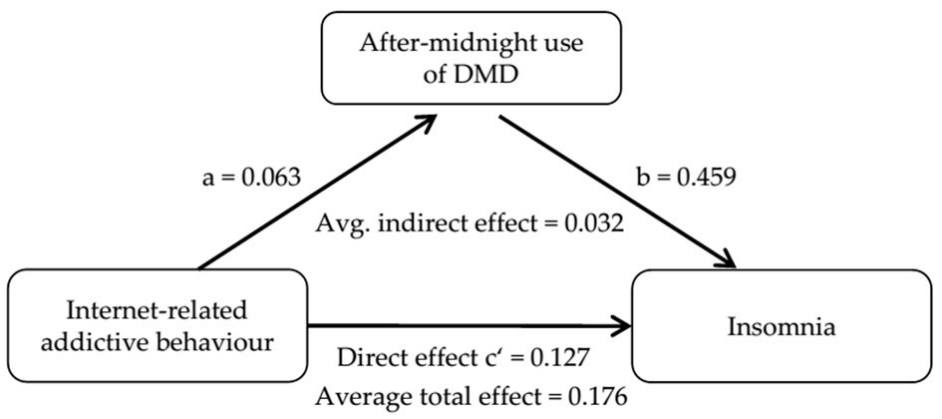
Direct, indirect and total effect of the mediation model. Values are unstandardized estimates and [95%-CI] of direct effects yielded from mediator and outcome model (see Table 5).

Table 5 shows a significant partial mediation of IRAB on sleep problems through after-midnight use of DMD [*b* = 0.032; 95%-CI (0.023; 0.040)]. The mediated proportion [0.182; 95%-CI (0.130; 0.250)] indicates that after-midnight use of DMD accounts for 18.2% of the total effect (see also Figure 4).

**Table 5:** Coefficients of mediation analysis measuring the association between IRAB and insomnia.

	**AFTMIDNGT**	**ISI**
**Direct effects**		
IRAB	0.063^***^ (0.047; 0.079)	0.127^***^ (0.083; 0.170)
AFTMIDNGT		0.459^***^ (0.329; 0.589)
**Average total effect**		
IRAB		0.176 (0.144; 0.210)
**Average indirect effect**		
IRAB through AFTMIDNGT		0.032 (0.023; 0.040)
Proportion mediated		0.182 (0.130; 0.250)

IRAB, Internet-related addictive behavior symptoms (CIUS-score); AFTMIDNGT, Count of days using DMD after midnight on evenings with subsequent school day (per school week); ISI, Insomnia symptoms (ISI-score).

Model is a mediation model with IRAB as predictor, count of days on using DMD after midnight on days with subsequent school day as mediator, insomnia symptoms as dependent measure and adjusted by school classes, anxiety symptoms, overall psychosocial constitution, age, gender and socioeconomic status.

Values are unstandardized estimates and (95%-CI) of direct effects yielded from mediator and outcome model. Average indirect and total effects are estimated using a quasi-Bayesian approximation as described in Imai et al. (46) based on 5,000 simulations.

^*^p < 0.05. ^**^p < 0.01. ^***^p < 0.001.

## 5 Discussion

Sleep problems among adolescents are an increasing concern as research indicates high prevalences of adolescents having insufficient and impaired sleep (47, 48) and also detrimental health and educational effects of sleep impairments relevant to adolescents. Among others, cognition (32) and school performance (49), immune system (50), mood states (51) and various aspects of risk behavior related to alcohol, drugs, sex, violence, transport (52) have been shown related to sleep impairment. Overexposure to digital media devices and internet applications has been shown in several studies, systematic reviews and meta-analyses to negatively affect sleep outcomes, especially if they are used during bedtime (11–14, 53, 54). The current study aimed to provide estimates about the extent of sleep impairments and after-midnight use of DMD on evenings with subsequent school day, which is unexplored in Styria, a major federal state of Austria. Besides, we wanted to test the hypothesis, that after-midnight use of DMD mediates the relation between internet-related addictive behavior and insomnia symptoms.

### 5.1 Insomnia and after-midnight use of DMD

In brief, we found that insomnia symptoms, IRAB, anxiety problems and an overall vulnerable psychosocial constitution are widespread among adolescents, which is in line with a recently published review (55), that concludes that mental health problems are “*considerably increasing in adolescent populations across many countries”* and that female adolescents show the greatest increases. In the current study, we also found that female adolescents are more likely to be affected by insomnia symptoms as a potential indicator of mental health problems. Nevertheless, it is important to notice, that not all positively screened pupils in this study are clinically relevant, since all prevalence estimates are just based on screenings. Diagnostic interviews were not conducted.

When considering bedtime routines, we clearly see that DMD are commonly used as a pre-sleep activity just before falling asleep. The vast majority uses DMD every single evening with subsequent school day as a pre-sleep activity. However, regression analysis showed that this routine is not associated with the extent of insomnia symptoms. This is an interesting finding, as it is not in line with the results yielded in literature reviews mentioned above (11–14). On the other hand, our results match with a recent sleep laboratory study in healthy young people with a mean age of 22.5 years focusing on social media use. The authors concluded their findings as follows: “*Social media use before sleep (controlling for effects of blue-light) had little effect on bedtime arousal and sleep quality than what was previously expected”* (56).

Although using DMD as a pre-sleep activity is not associated with insomnia symptoms in the present study, we found, that this habit is significantly related to after-midnight use of DMD, which in turn is significantly associated with insomnia symptoms. However, it remains uncertain, whether after-midnight use of DMD is solely linked to insomnia due to shortened sleep times, or whether there are evidenced aspects such as light exposure (57, 58), as well as cognitive and emotional arousal associated for example with social media use (59) that were not addressed in this study.

Additionally, after-midnight use of DMD partially mediates the association between IRAB and insomnia suggesting that adolescents showing IRAB are more likely to engage with DMD late at night, which contributes to disrupted sleep patterns. Using DMD during bedtime may extend usage time beyond midnight. After-midnight use of DMD on usually more than one evening with subsequent school day during a regular school week is associated with a predicted insomnia score that exceeds the threshold for impaired sleep. Considering the extent of the occurrence of after-midnight use of DMD among adolescents (on average on 1.7 evenings per school week), this finding clearly mandates respective policy planning related to information and sensitization among adolescents themselves, and involving parents as well.

Parents do act as role models for various (health) behavioral aspects, which is, fortunately, no warranty that their children act the same, but there is evidence of increased likelihoods [e.g., for physical activity (60), smoking (61) and nutrition (62)]. This also applies for sleep hygiene, DMD-related bedtime routines and the general amount of DMD-usage (23, 63). Considering parents, it is interesting that parental rules are not associated with the extent of after midnight use of DMD. This may be due to a measurement issue, since the presence of parental rules concerning usage times of DMD was surveyed in a general way and not specifically related to bedtime use. On the other hand, another publication based on the present data showed, that parental regulative rules are positively associated with IRAB (63). Taking the specific coding into account, that means that adolescents subjected to parental rules show higher extent of IRAB. This suggests that rules mainly come into play as an interventional, rather than as a preventive measure.

The findings also show that the extent of insomnia symptoms is negatively associated with individual's SES (the less SES, the more symptoms), when controlling for behavioral factors (using DMD as a pre-sleep activity or after midnight) and aspects of mental health such as anxiety and psychosocial constitution. This is in line with reviews addressing sleep inequalities between different populations (64, 65) and supports the demand of policies (66) e.g., framed in the socioecological model of sleep (67, 68), which states that, in addition to aspects at individual level, other layers in which individual aspects are embedded, account for sleep disparities. These layers are the adolescents' immediate local physical sleep environment (e.g., crowded, noisy), which in turn is shaped by community based aspects (traffic and noise, light, green space) and general societal aspects (school starting times, working conditions). Adolescents with lower SES are more likely to experience adverse conditions in their physical sleep environment and community-based aspects of sleep. Therefore, sleep-promoting (or generally health-promoting) policies that focus exclusively on individual lifestyle-related aspects fall short and must be supplemented by the aforementioned macro-aspects, e.g., in urban planning, public housing, etc.

### 5.2 Strengths and limitations

A major strength of this study is that it provides an evidence-based foundation for policy planning in Austria, which, although strongly demanded by multiple sectors, was limited by a lack of reliable data until now. Another strength is the study's sampling strategy, as well as its high participation rate and large sample size yielding population-representative data and therefore enables generalizability of the estimated prevalences. Furthermore, well-established instruments were used.

However, information on physiological and environmental aspects of sleep, as well as chronotypes, was not collected in the survey. The regression model applied for insomnia as outcome variable accounts for 36% of explained variance, clearly indicating that there are uncovered aspects of insomnia, such as physiological, environmental and societal aspects.

Second, another limitation of this study is its cross-sectional nature, limiting causal inference due to unknown temporal ordering of the observed measures. Since there is evidence of bidirectionality of the association of dysfunctional digital media use and sleep health (15), future longitudinal studies should take this into account when investigating the relation between late-night use of DMD and insomnia symptoms. The same applies for the relation between IRAB and insomnia symptoms.

Third, the used data-set contains no information about the content (social media activity, playing games, etc.) of after-midnight use of DMD, which probably would provide some explanation of the association of after-midnight use of DMD and insomnia symptoms, as indicated in a recent meta-analysis (15).

Fourth, although the data contains information whether parents established rules related to general usage times, we do not know to what extent these parental regulations also apply for usage behavior in the evening and during bedtime and to what extent they are controlled. Further research should therefore examine this aspect in a more differentiated way.

Finally, the data is solely based on self-report measures. Although this kind of data acquisition is widely used in social science literature, it is prone to be biased due to under- and over-reporting in similar proportions, as shown in a recent meta-analysis of discrepancies between logged and self-reported digital media use (69). The same discordance of objective (e.g., actigraphy) and self-report measurements was shown for sleep duration in a very specific target group of individuals with chronic kidney disease (70).

## 6 Conclusion

This study aimed to provide estimates about the extent of sleep impairments and after-midnight use of DMD in a major federal state of Austria, as already requested by practitioners in health care and education. The results show high prevalences not only related to insomnia, bedtime and late-night use of DMD, but also for other mental impairments such as IRAB, anxiety and the overall psychosocial constitution of Styrian adolescents. After-midnight use of DMD is clearly associated with insomnia symptoms and with IRAB. The average extent of after-midnight use of DMD is higher than what is expected for unimpaired sleep. The association between IRAB and insomnia is partially mediated by after-midnight use of DMD. This should be taken into account in the treatment of internet use disorder and in early interventions. In conclusion, the findings demonstrate a clear need for policy planning and recognition of the issue as a public health concern. At the level of universal prevention existing programs to strengthen life skills among children and adolescents could increase efforts to promote self-control in dealing with DMD. This issue may also be addressed in the subject “Digital Education”, which has already been implemented permanently in Austrian schools. Adolescents and parents should be aware that late-night use of DMD can impair sleep. Parents should therefore be encouraged to monitor their children's bedtime usage patterns discreetly, implement preventive age-appropriate usage rules and ensure their adherence. Furthermore, longitudinal studies are required to allow for a better mapping of causal effects.

## Data availability statement

The data analyzed in this study is subject to the following licenses/restrictions: the data used in this publication originate from a commissioned work assigned by Gesundheitsfonds Steiermark as the owner of the dataset. Data is available on request after authorization from Gesundheitsfonds Steiermark. Requests to access these datasets should be directed to Juliane Cichy, juliane.cichy@gfstmk.at.

## Ethics statement

The studies involving humans were approved by Ethics Committee of the Medical University of Graz (EC-number: 33-600 ex 20/21). The studies were conducted in accordance with the local legislation and institutional requirements. Written informed consent for participation was not required from the participants or the participants' legal guardians/next of kin because consent was provided by the Styrian directorate of education and by the schools' principals. The parents of the school students received an information letter about the survey with the option of refusing to participate. School students had to provide informed consent before the survey could start.

## Author contributions

TL-H: Conceptualization, Formal analysis, Methodology, Visualization, Writing – original draft. KM: Conceptualization, Writing – review & editing. MP: Formal analysis, Writing – review & editing. ES: Methodology, Writing – review & editing. EG: Writing – review & editing. WF: Conceptualization, Writing – review & editing.

## References

[1] Integral. AIM. - Austrian Internet Monitor. Wien: Integral (2023).

[2] Südwest MF. JIM-Studie 2023. Stuttgart: Jugend, Information, Medien (2023).

[3] Young K. Caught in the Net: Understanding Internet Addiction. New York, NY: Wiley (1998).

[4] Aarseth E Bean AM Boonen H Colder Carras M Coulson M Das D. Scholars' open debate paper on the World Health Organization ICD-11 Gaming Disorder proposal. J Behav Addict. (2017) 6:267–70. 10.1556/2006.5.2016.088 28033714 PMC5700734

[5] Dullur P Starcevic V. Internet gaming disorder does not qualify as a mental disorder. Aust N Z J Psychiatry. (2018) 52:110–1. 10.1177/0004867417741554 29119800

[6] van Rooij AJ Ferguson CJ Colder Carras M Kardefelt-Winther D Shi J Aarseth E. A weak scientific basis for gaming disorder: Let us err on the side of caution. J Behav Addict. (2018) 7:1–9. 10.1556/2006.7.2018.19 29529886 PMC6035022

[7] Pan YC Chiu YC Lin YH. Systematic review and meta-analysis of epidemiology of internet addiction. Neurosci Biobehav Rev. (2020) 118:612–22. 10.1016/j.neubiorev.2020.08.013 32853626

[8] Werner AM Petersen J Müller KW Tibubos AN Schäfer M Mülder LM. Prävalenz von Internetsucht vor und während der COVID-19 Pandemie unter Studierenden der Johannes Gutenberg-Universität Mainz. Suchttherapie. (2021) 22:183–93. 10.1055/a-1653-8186

[9] Neumann I Lindenberg K. Internetnutzungsstörungen unter deutschen Jugendlichen vor und während der COVID-19-Pandemie. Kindheit Entwicklung. (2022) 31:193–9. 10.1026/0942-5403/a000390

[10] Hale L Kirschen GW LeBourgeois MK Gradisar M Garrison MM Montgomery-Downs H. Youth screen media habits and sleep: sleep-friendly screen behavior recommendations for clinicians, educators, and parents. Child Adolesc Psychiatr Clin N Am. (2018) 27:229–45. 10.1016/j.chc.2017.11.014 29502749 PMC5839336

[11] Hale L Guan S. Screen time and sleep among school-aged children and adolescents: a systematic literature review. Sleep Med Rev. (2015) 21:50–8. 10.1016/j.smrv.2014.07.007 25193149 PMC4437561

[12] de Sa S Baiao A Marques H Marques MDC Reis MJ Dias S. The influence of smartphones on adolescent sleep: a systematic literature review. Nurs Rep. (2023) 13:612–21. 10.3390/nursrep13020054 37092482 PMC10123719

[13] Brautsch LA Lund L Andersen MM Jennum PJ Folker AP Andersen S. Digital media use and sleep in late adolescence and young adulthood: a systematic review. Sleep Med Rev. (2022) 68:101742. 10.1016/j.smrv.2022.101742 36638702

[14] Lund L Sølvhøj IN Danielsen D Andersen S. Electronic media use and sleep in children and adolescents in western countries: a systematic review. BMC Public Health. (2021) 21:1598. 10.1186/s12889-021-11640-9 34587944 PMC8482627

[15] Pagano M Bacaro V Crocetti E. “Using digital media or sleeping … that is the question”. A meta-analysis on digital media use and unhealthy sleep in adolescence. Comp Hum Behav. (2023) 146:107813. 10.1016/j.chb.2023.107813

[16] Alimoradi Z Lin CY Brostrom A Bulow PH Bajalan Z Griffiths MD. Internet addiction and sleep problems: a systematic review and meta-analysis. Sleep Med Rev. (2019) 47:51–61. 10.1016/j.smrv.2019.06.004 31336284

[17] Li Y Li G Liu L Wu H. Correlations between mobile phone addiction and anxiety, depression, impulsivity, and poor sleep quality among college students: a systematic review and meta-analysis. J Behav Addict. (2020) 9:551–71. 10.1556/2006.2020.00057 32903205 PMC8943681

[18] Zhang J Zhang X Zhang K Lu X Yuan G Yang H. An updated of meta-analysis on the relationship between mobile phone addiction and sleep disorder. J Affect Disord. (2022) 305:94–101. 10.1016/j.jad.2022.02.008 35149136

[19] Nalwa K Anand AP. Internet addiction in students: a cause of concern. Cyberpsychol Behav. (2003) 6:653–6. 10.1089/109493103322725441 14756932

[20] Lee S-J. Parental restrictive mediation of children's internet use: effective for what and for whom? New Media Soc. (2013) 15:466–81. 10.1177/146144481245241226075919

[21] Lukavska K Hrabec O Lukavsky J Demetrovics Z Kiraly O. The associations of adolescent problematic internet use with parenting: a meta-analysis. Addict Behav. (2022) 135:107423. 10.1016/j.addbeh.2022.107423 35933287

[22] Kalmus V Blinka L Ólafsson K. Does it matter what mama says: evaluating the role of parental mediation in European adolescents' excessive internet use. Child Soc. (2015) 29:122–33. 10.1111/chso.12020

[23] Buxton OM Chang AM Spilsbury JC Bos T Emsellem H Knutson KL. Sleep in the modern family: protective family routines for child and adolescent sleep. Sleep Health. (2015) 1:15–27. 10.1016/j.sleh.2014.12.002 26779564 PMC4712736

[24] Hirshkowitz M Whiton K Albert SM Alessi C Bruni O DonCarlos L. National Sleep Foundation's sleep time duration recommendations: methodology and results summary. Sleep Health. (2015) 1:40–3. 10.1016/j.sleh.2014.12.010 29073412

[25] Paruthi S Brooks LJ D'Ambrosio C Hall WA Kotagal S Lloyd RM. Consensus statement of the american academy of sleep medicine on the recommended amount of sleep for healthy children: methodology and discussion. J Clin Sleep Med. (2016) 12:1549–61. 10.5664/jcsm.6288 27707447 PMC5078711

[26] Correa VS Centofanti S Dorrian J Wicking A Wicking P Lushington K. The effect of mobile phone use at night on the sleep of pre-adolescent (8-11 year), early adolescent (12-14 year) and late adolescent (15-18 year) children: a study of 252,195 Australian children. Sleep Health. (2022) 8:277–82. 10.1016/j.sleh.2022.02.004 35400615

[27] Roane BM Taylor DJ. Adolescent insomnia as a risk factor for early adult depression and substance abuse. Sleep. (2008) 31:1351–6. 18853932 PMC2572740

[28] Scott J Kallestad H Vedaa O Sivertsen B Etain B. Sleep disturbances and first onset of major mental disorders in adolescence and early adulthood: a systematic review and meta-analysis. Sleep Med Rev. (2021) 57:101429. 10.1016/j.smrv.2021.101429 33549912

[29] McMakin DL Alfano CA. Sleep and anxiety in late childhood and early adolescence. Curr Opin Psychiatry. (2015) 28:483–9. 10.1097/YCO.0000000000000204 26382163 PMC4670558

[30] Chellappa SL Aeschbach D. Sleep and anxiety: from mechanisms to interventions. Sleep Med Rev. (2022) 61:101583. 10.1016/j.smrv.2021.101583 34979437

[31] Fernandez-Mendoza J Bourchtein E Calhoun S Puzino K Snyder CK He F. Natural history of insomnia symptoms in the transition from childhood to adolescence: population rates, health disparities, and risk factors. Sleep. (2021) 44:zsaa187. 10.1093/sleep/zsaa187 32929504 PMC7953218

[32] Yang FN Xie W Wang Z. Effects of sleep duration on neurocognitive development in early adolescents in the USA: a propensity score matched, longitudinal, observational study. Lancet Child Adolesc Health. (2022) 6:705–12. 10.1016/S2352-4642(22)00188-2 35914537 PMC9482948

[33] Medic G Wille M Hemels ME. Short- and long-term health consequences of sleep disruption. Nat Sci Sleep. (2017) 9:151–61. 10.2147/NSS.S134864 28579842 PMC5449130

[34] Bruni O DelRosso LM Mogavero MP Angriman M Ferri R. Chronic insomnia of early childhood: Phenotypes and pathophysiology. Neurosci Biobehav Rev. (2022) 137:104653. 10.1016/j.neubiorev.2022.104653 35398115

[35] Levenson JC Thoma BC Hamilton JL Choukas-Bradley S Salk RH. Sleep among gender minority adolescents. Sleep. (2021) 44:zsaa185. 10.1093/sleep/zsaa185 32949142 PMC7953209

[36] Gerber M Lang C Lemola S Colledge F Kalak N Holsboer-Trachsler E. Validation of the German version of the insomnia severity index in adolescents, young adults and adult workers: results from three cross-sectional studies. BMC Psychiatry. (2016) 16:174. 10.1186/s12888-016-0876-8 27245844 PMC4888604

[37] Bischof G Bischof A Besser B Rumpf HJ. Problematische und pathologische Internetnutzung: Entwicklung eines Kurzscreenings (PIEK). Abschlussbericht an das Bundesministerium für Gesundheit Universität zu Lübeck, Klinik für Psychiatrie und Psychotherapie. Berlin: Bundesministerium für Gesundheit (2016).

[38] Besser B Rumpf HJ Bischof A Meerkerk GJ Higuchi S Bischof G. Internet-related disorders: development of the short compulsive internet use scale. Cyberpsychol Behav Soc Netw. (2017) 20:709–17. 10.1089/cyber.2017.0260 29125788

[39] Meerkerk GJ Van Den Eijnden RJ Vermulst AA Garretsen HF. The Compulsive Internet Use Scale (CIUS): some psychometric properties. Cyberpsychol Behav. (2009) 12:1–6. 10.1089/cpb.2008.0181 19072079

[40] Lowe B Decker O Muller S Brahler E Schellberg D Herzog W. Validation and standardization of the Generalized Anxiety Disorder Screener (GAD-7) in the general population. Med Care. (2008) 46:266–74. 10.1097/MLR.0b013e318160d093 18388841

[41] Mossman SA Luft MJ Schroeder HK Varney ST Fleck DE Barzman DH. The Generalized Anxiety Disorder 7-item scale in adolescents with generalized anxiety disorder: signal detection and validation. Ann Clin Psychiatry. (2017) 29:227–34. 29069107 PMC5765270

[42] Klasen H Woerner W Rothenberger A Goodman R. Die deutsche Fassung des strengths and difficulties questionnaire (SDQ-Deu)-Übersicht und Bewertung erster Validierungs-und Normierungsbefunde (2003). 14526759

[43] Tingley D Yamamoto T Hirose K Keele L Imai K. mediation: R package for causal mediation analysis. J Stat Softw. (2014) 59:1–38. 10.18637/jss.v059.i0526917999

[44] Gelman A. Scaling regression inputs by dividing by two standard deviations. Stat Med. (2008) 27:2865–73. 10.1002/sim.3107 17960576

[45] Nakagawa S Schielzeth H. A general and simple method for obtaining R2 from generalized linear mixed-effects models. Methods Ecol Evol. (2013) 4:133–42. 10.1111/j.2041-210x.2012.00261.x30239975

[46] Imai K Keele L Tingley D. A general approach to causal mediation analysis. Psychol Methods. (2010) 15:309–34. 10.1037/a0020761 20954780

[47] Gariepy G Danna S Gobina I Rasmussen M Gaspar de Matos M Tynjälä J. How are adolescents sleeping? Adolescent sleep patterns and sociodemographic differences in 24 European and North American Countries. J Adolesc Health. (2020) 66(6, Suppl.):S81–8. 10.1016/j.jadohealth.2020.03.013 32446613

[48] Gradisar M Gardner G Dohnt H. Recent worldwide sleep patterns and problems during adolescence: a review and meta-analysis of age, region, and sleep. Sleep Med. (2011) 12:110–8. 10.1016/j.sleep.2010.11.008 21257344

[49] Perez-Lloret S Videla AJ Richaudeau A Vigo D Rossi M Cardinali DP. A multi-step pathway connecting short sleep duration to daytime somnolence, reduced attention, and poor academic performance: an exploratory cross-sectional study in teenagers. J Clin Sleep Med. (2013) 09:469–73. 10.5664/jcsm.2668 23674938 PMC3629321

[50] Orzech KM Acebo C Seifer R Barker D Carskadon MA. Sleep patterns are associated with common illness in adolescents. J Sleep Res. (2014) 23:133–42. 10.1111/jsr.12096 24134661 PMC4115328

[51] Booth SA Carskadon MA Young R Short MA. Sleep duration and mood in adolescents: an experimental study. Sleep. (2020) 44:zsaa253. 10.1093/sleep/zsaa253 33245773

[52] Short MA Weber N. Sleep duration and risk-taking in adolescents: a systematic review and meta-analysis. Sleep Med Rev. (2018) 41:185–96. 10.1016/j.smrv.2018.03.006 29934128

[53] Pillion M Gradisar M Bartel K Whittall H Kahn M. What's “app”-ning to adolescent sleep? Links between device, app use, and sleep outcomes. Sleep Med. (2022) 100:174–82. 10.1016/j.sleep.2022.08.004 36084495

[54] Carter B Rees P Hale L Bhattacharjee D Paradkar MS. Association between portable screen-based media device access or use and sleep outcomes: a systematic review and meta-analysis. JAMA Pediatr. (2016) 170:1202–8. 10.1001/jamapediatrics.2016.2341 27802500 PMC5380441

[55] Keyes KM Platt JM. Annual research review: sex, gender, and internalizing conditions among adolescents in the 21st century - trends, causes, consequences. J Child Psychol Psychiatry. (2023) 65:384–407. 10.1111/jcpp.13864 37458091 PMC12341061

[56] Combertaldi SL Ort A Cordi M Fahr A Rasch B. Pre-sleep social media use does not strongly disturb sleep: a sleep laboratory study in healthy young participants. Sleep Med. (2021) 87:191–202. 10.1016/j.sleep.2021.09.009 34627122

[57] Chinoy ED Duffy JF Czeisler CA. Unrestricted evening use of light-emitting tablet computers delays self-selected bedtime and disrupts circadian timing and alertness. Physiol Rep. (2018) 6:e13692. 10.14814/phy2.13692 29845764 PMC5974725

[58] Crowley SJ Cain SW Burns AC Acebo C Carskadon MA. Increased sensitivity of the circadian system to light in early/mid-puberty. J Clin Endocrinol Metab. (2015) 100:4067–73. 10.1210/jc.2015-2775 26301944 PMC4702443

[59] Scott H Woods HC. Understanding links between social media use, sleep and mental health: recent progress and current challenges. Curr. Sleep Med Rep. (2019) 5:141–9. 10.1007/s40675-019-00148-9

[60] Erkelenz N Kobel S Kettner S Drenowatz C Steinacker JM. Parental activity as influence on children‘s BMI percentiles and physical activity. J Sports Sci Med. (2014) 13:645–50. 25177194 PMC4126304

[61] Kandel DB Griesler PC Hu M-C. Intergenerational patterns of smoking and nicotine dependence among US adolescents. American Journal of Public Health. (2015) 105:e63–72. 10.2105/AJPH.2015.302775 26378847 PMC4605183

[62] Draxten M Fulkerson JA Friend S Flattum CF Schow R. Parental role modeling of fruits and vegetables at meals and snacks is associated with children's adequate consumption. Appetite. (2014) 78:1–7. 10.1016/j.appet.2014.02.017 24630934 PMC4034448

[63] Lederer-Hutsteiner T Freidl W. Prevalence and Correlates of Internet-Related Addictive Behaviour Among Styrian Pupils. Graz: Doc-Day of the Medical University of Graz (2024).

[64] Grandner MA. Sleep, health, and society. Sleep Med Clin. (2017) 12:1–22. 10.1016/j.jsmc.2016.10.012 28159089 PMC6203594

[65] Hale L Troxel W Buysse DJ. Sleep health: an opportunity for public health to address health equity. Annu Rev Public Health. (2020) 41:81–99. 10.1146/annurev-publhealth-040119-094412 31900098 PMC7944938

[66] Lim DC Najafi A Afifi L Bassetti CLA Buysse DJ Han F. The need to promote sleep health in public health agendas across the globe. The Lancet Public Health. (2023) 8:e820–e6. 10.1016/S2468-2667(23)00182-2 37777291 PMC10664020

[67] Grandner MA. Chapter 5 - Social-ecological model of sleep health. In: Grandner MA, editor. Sleep and Health. Academic Press; Elsevier Inc. (2019). p. 45−53.

[68] Prather AA. Waking up to the importance of sleep: opportunities for policy makers. Policy Insights Behav Brain Sci. (2023) 10:25–32. 10.1177/23727322221144651

[69] Parry DA Davidson BI Sewall CJR Fisher JT Mieczkowski H Quintana DS. systematic review and meta-analysis of discrepancies between logged and self-reported digital media use. Nat Hum Behav. (2021) 5:1535–47. 10.1038/s41562-021-01117-5 34002052

[70] Carvalho KSB Lauar JC Drager LF Moyses RMA Elias RM. Self-reported and objective sleep duration in patients with CKD: are they telling the same story? J Bras Nefrol. (2023) 45:102–5. 10.1590/2175-8239-jbn-2022-0015pt 35993531 PMC10139718

